# A Rapid Protocol of Crude RNA/DNA Extraction for RT-qPCR Detection and Quantification of '*Candidatus* Phytoplasma prunorum'

**DOI:** 10.1371/journal.pone.0146515

**Published:** 2016-01-07

**Authors:** Stefano Minguzzi, Federica Terlizzi, Chiara Lanzoni, Carlo Poggi Pollini, Claudio Ratti

**Affiliations:** DipSA - Patologia Vegetale, Università di Bologna, Viale G. Fanin, 40–40127, Bologna, Italy; Julius Kuehn-Institute (JKI), GERMANY

## Abstract

Many efforts have been made to develop a rapid and sensitive method for phytoplasma and virus detection. Taking our cue from previous works, different rapid sample preparation methods have been tested and applied to *Candidatus* Phytoplasma prunorum (‘*Ca*. P. prunorum’) detection by RT-qPCR. A duplex RT-qPCR has been optimized using the crude sap as a template to simultaneously amplify a fragment of 16S rRNA of the pathogen and 18S rRNA of the host plant. The specific plant 18S rRNA internal control allows comparison and relative quantification of samples. A comparison between DNA and RNA contribution to qPCR detection is provided, showing higher contribution of the latter. The method presented here has been validated on more than a hundred samples of apricot, plum and peach trees. Since 2013, this method has been successfully applied to monitor ‘*Ca*. P. prunorum’ infections in field and nursery. A triplex RT-qPCR assay has also been optimized to simultaneously detect ‘*Ca*. P. prunorum’ and *Plum pox virus* (PPV) in *Prunus*.

## Introduction

Phytoplasmas are uncultured wall-less bacteria (class *Mollicutes*), which live in the phloem of their host plants and are transmitted by insect vectors belonging to the *Homoptera* order [1]. These microorganisms are associated with more than 300 diseases in several hundred plant species worldwide [2]. At present, phytoplasmas are subdivided into 15 groups based on the similarity of their 16S ribosomal DNA (rDNA) sequences [3]. The apple proliferation group (16SrX) includes quarantine species responsible for major economic losses in Europe, such as ‘*Ca*. P. pyri’ associated with pear decline (PD), ‘*Ca*. P. prunorum’ associated with European stone fruit yellows (ESFY), ‘*Ca*. P. mali’ associated with apple proliferation (AP) and peach yellow leafroll (PYLR). [4, 5].

‘*Ca*. P. prunorum’ generally induces yellows, tree decline or die-back and vegetative disorders with typical symptoms such as an early bud break and leaf rolling in most wild and cultivated *Prunus* species [6]. ESFY is mainly known in Europe, but has also been reported in Turkey and Azerbaijan [5] and it was first described as a decline of the Japanese plum (*Prunus salicina*) in Italy [7]. ‘*Ca*. P. prunorum’ causes substantial economic loss due to the decline and death of the infected trees. ‘*Ca*. P. prunorum’ can spread rapidly in stone fruit cultivating areas thanks to its highly efficient natural vector *Cacopsilla pruni* and the agricultural use of non-symptomatic infected plant material. In apricot orchards, the number of infected trees can double in few years [8].

The symptomatic detection of ‘*Ca*. P. prunorum’ is unreliable because of the presence of asymptomatic infected plants and the symptom variability among host plants and seasons [5]. Therefore, in order to reduce the spread of ESFY, a specific, sensitive molecular diagnostic method is required for an early identification and removal of infected plants.

So far, several molecular assays have been published based on nested-PCR or SYBR Green qPCR methods. Previous works reported nested-PCR protocols based on two step amplification of the 16S/23S rRNA gene [9, 10] or the aceF gene [11]. Although the introduction of a second round of amplification allows a more specific detection of phytoplasma, it increases the risk of false positives due to cross contamination. SYBR Green-based qPCR protocols have been designed for 16SrX group phytoplasmas detection [4] and for ‘*Ca*. P. prunorum’ specific detection in plants and *C*. *pruni* vector [12].

A protocol for the detection of three phytoplasmas of group X including *‘Ca*. P. prunorum’ by qPCR has previously been reported [13]. This method, as all the previously reported protocols, is based on DNA amplification, although previous works on Flavescence dorée phytoplasma detection showed a higher sensitivity by adding a reverse transcriptase (RT) step for RNA amplification [14, 15]. Up to now, no TaqMan^®^ based RT-qPCR protocol for ‘*Ca*. P. prunorum’ detection has been published.

To evaluate a detection protocol based on PCR amplification, the nucleic acid extraction method must be carefully selected. Several commercial RNA and DNA purification kits are available [16]. On the one hand, these kits are quite rapid and efficient; on the other hand, they are expensive and strongly increase final detection costs. Nevertheless, affordable extraction protocols were developed by using chemical compounds such as cetyltrimethyl ammonium bromide (CTAB) [17, 18]. These protocols have been shown to be as efficient as commercial kits but they have the main disadvantage of being time-consuming. Consequently, many attempts to avoid nucleic acid extraction and to develop rapid sample preparation protocol have been made [15, 19–22]. Results from the previous work showed different efficient ways to perform PCR amplification after rapid crude sap preparation. These data also showed that method efficiency depends on the pathogen and plant host, presenting a wide variability even between different virus and phytoplasma strains within the same plant host species [23].

In this manuscript, we have developed a rapid and inexpensive crude sap extraction method, which exploits Nylon membrane discs and can be applied to a new TaqMan^®^ based RT-qPCR protocol for specific 16S rRNA amplification of ‘*Ca*. P. prunorum’. By simultaneously using internal control primers and probe for plant 18S rRNA, we optimized a highly sensitive protocol, suitable for large scale analysis, which also allows relative quantification of phytoplasma in the host tissues. The method presented here has been successfully validated for the detection of ‘*Ca*. P. prunorum’ on more than one hundred samples of apricot, plum and peach trees. Furthermore, storage property of nylon membrane discs has also been evaluated over a period of 4 months. Finally, a triplex RT-qPCR assay has been developed to simultaneously detect ‘*Ca*. P. prunorum’ and PPV in *Prunus*.

## Materials and Methods

Each sample collected and used in the present work has been obtained under the permission of the responsible authority. In particular, the Plant Protection Service of the Emilia-Romagna Region for samples collected from the province of Forlì-Cesena, the Consorzio Fitosanitario Provinciale of Modena for samples from the Modena province and the Plant Protection Service of “Provincia Autonoma di Trento” for samples collected from the province of Trento. We also confirm that the study did not involve endangered or protected species.

### Sources of phytoplasmas and plant material preparation

The rapid method for phytoplasma detection was developed on sap extracted from ESFY-inoculated apricot plants (*Prunus armenica*) from the authors’ collection. As well, AP-infected apple samples, PD-infected pear samples, bois noir and flavescence dorée-infected grape samples, used to evaluate cross reaction signal, belong to the authors’ collection. The PPV-infected sample used for triplex RT-qPCR optimisation has been collected and managed in agreement with the Plant Protection Service of Emilia-Romagna Region. The 40 ESFY-positive plum samples, employed to compare the spot method coupled with RT-qPCR with CTAB method coupled with qPCR, have been collected in orchards located in the province of Modena (Italy). The spot method was evaluated in routine conditions on 62 plant samples from apricot, peach and plum trees showing ESFY symptoms coming from the provinces of Forlì-Cesena and Trento (Italy).

The plant material was collected under appropriate conditions to prevent cross-contamination. All the samples used in both CTAB and rapid DNA/RNA sample preparation consisted of 1 g of phloem-rich tissue, collected after the removal of the bark from woody shoots of the fruit trees, placed in extraction bags (Bioreba). Samples were immediately tested or stored at -20°C until use.

### DNA/RNA CTAB extraction and rapid sample preparation methods

DNA/RNA extraction was performed using a CTAB method [24], resuspending the nucleic acids in 1mL of nuclease-free Milli-Q water.

Samples for the rapid preparation method were homogenized with a drill press in different volumes of a grinding buffer (15 mM Na_2_CO_3_, 34.9 mM NaHCO_3_, 2% polyvinyl-pyrrolidone [PVP]- 40, 0.2% bovine serum albumin [BSA], 0.05% Tween 20 and 1% Na_2_S_2_O_5_, pH 9.6) in order to have final dilutions of 1:5, 1:10, 1:20 (w/v) [15, 23, 25]. Sap obtained was used in different rapid template preparation protocols previously reported [19–21, 23]. Six mm diameter discs were cut from nylon membrane (GE Healthcare Life Sciences) using a standard hole puncher. Five μl of sap were spotted onto nylon membrane discs, placed inside 0.2 mL microtubes, and dried under vacuum conditions for 10 min (spot method). Alternatively, 5 μL of the extract were directly loaded into microtubes (dilution method). All the samples were boiled for 10 min after the addition of 50 or 100 μL of four different resuspension solutions: 0,5% Triton X-100, GES (0.1 M glycine, 0.05 M NaCl, 1 mM EDTA, pH 8), GES with the addition of 0,5% Triton X-100, GES- Polyvinylpolypyrrolidone [PVPP] (GES with hydrated insoluble PVPP in a ratio 1:1 (v:v); PVPP was previously hydrated by soaking 8 g for at least 2 h in 250 ml of GES buffer and excess solution was removed after overnight decantation) and GES-PVPP with the addition of 0,5% Triton X-100.

After boiling, samples obtained through above methods were immediately chilled on ice and centrifuged at 16.000 g for 2 min at 4°C. Finally, 2 μl of supernatant were used as template for the duplex RT-qPCR described below. Technical duplicates were performed in all the experiment for the validation of the duplex assay.

In the time course experiment, samples were prepared and tested using optimized conditions (see Results). Five μl of crude sap were prepared on 6 mm diameter nylon membrane discs as described above and immediately tested by RT-qPCR or stored at either 4°C or room temperature. Stored spotted samples along with the corresponding frozen crude sap stored at -20°C were tested in triplicate by RT-qPCR after 2 and 4 months using the optimized spot method (see Results).

For the triplex optimization, one sample infected by ESFY and one sample infected by PPV were prepared with the spot method and mixed in a ratio 1:1. Two μl of supernatant were used as template for the duplex RT-qPCR described below.

### Probes and primers design

All the primers and probes employed in this work were designed using Primer Express 2.0 software (Life Technologies).

A specific ‘*Ca*. P. prunorum’ primer and MGB probe assay (Table 1) was designed using a previously reported sequence alignment of the 16S rRNA gene region of nine different phytoplasma strains belonging to six distinct 16S rRNA subclades [25]. Accession numbers of 16S rRNA genes used for the alignment were the following: ESFY (AY029540), AP (AF248958), PD (Y168952), Stolbur (AF248959), Aster yellows (AF503568), Elm yellows (X68376), Flavescences dorée (X76560), Bermuda grass white leaf (Y16388), Italian clover phyllody (X77482). The amplicon length of the ESFY 16S assay was 74 nucleotides. A plant DNA and RNA control assay was designed using the sequence of 18S ribosomal RNA. Previously published primer and probe sequences [26], specific for many plant species including grape (AF321271.1), strawberry (X15590), citrus (U38312), prunus (L28749), soybean (AH001585) and barley (AH001585), were slightly modified into a MGB based probe assay named DiSTA 18S (Table 1). Amplicon length of DiSTA 18S was 61 nucleotides.

**Table 1:** List of primers and probes used for detection of *Ca*. P. prunorum, endogenous control for host plant (Plants) and PPV.

Name	Primer / Probe	Specificity	Sequence 5'-3'
ESFY 16S-F	Forward	Ca. P. prunorum	CGA ACG GGT GAG TAA CAC GTA A
ESFY 16S-R	Reverse	Ca. P. prunorum	CCA GTC TTA GCA GTC GTT TCC A
ESFY 16S	Probe	Ca. P. prunorum	FAM-TAA CCT GCC TCT CAG GCG-MGB
DiSTA 18S-F	Forward	Plants	TGA CGG AGA ATT AGG GTT CGA
DiSTA 18S-R	Reverse	Plants	CTT GGA TGT GGT AGC CGT TTC
DiSTA 18S	Probe	Plants	NED-CGG AGA GGG AGC CTG-MGB
P316D	Forward	PPV	GAT TAA CAT CAC CAG CGG TGT G
P316M	Forward	PPV	GAT TCA CGT CAC CAG CGG TGT G
P241	Reverse	PPV	CGT TTA TTT GGC TTG GAT GGA A
PPV-DM	Probe	PPV	VIC-CGT CGG AAC ACA AGA AGA GGA CAC AGA-TAMRA

The previously published PPV specific primers and probe validated in a previous work were used in the triplex RT-qPCR assay in the present work [27] (Table 1). A TAMRA quencher has been maintained in PPV-DM probe as reported in the original paper [27].

Fluorogenic probes labelled at the 5’ with FAM (ESFY 16S), NED (DiSTA 18S) or VIC (PPV-DM) were purchased from Applied Biosystems (Table 1).

### Duplex and triplex RT-qPCR, Relative Quantification and qPCR Efficiency

The optimal primer concentrations of each target (‘*Ca*. P. prunorum’, PPV and endogenous control 18S rRNA) were determined in separate tubes by running a matrix of forward and reverse primer concentrations according to Applied Biosystems user bulletin no. 5 (P/N 4306236B; http://www.appliedbiosystems.com). The subsequent multiplex optimisation was carried out by running several matrices of different concentrations of the three sets of primers and probes. The following additives at different concentrations were also tested separately to improve the reaction: DMSO (0.5%-1%-2.5%-5%), ammonium sulphate (5mM-10mM-15mM), formamide (0.5%-1%-2%-5%), betaine (1M-1.5M-3M-5M), TritonX 100 (0.25%-0.5%-1%), DTT (0.5mM-1mM-1.5mM), glycerol (2.5%-5%-10%), BSA (0.5 mg/ml). Finally, different concentrations of RT-qPCR reagents were tested: MgCl_2_ (4.4mM-5.5mM-8mM), M-MLV Reverse Transcriptase (Promega) (2U-4U-8U-10U), AmpliTaq Gold DNA Polymerase (Life Technologies) (0.5U-1.25U-2U).

The duplex RT-qPCR reaction was performed in a total volume of 25 μL containing 2 μL of template nucleic acid, 1X Buffer A (Life Technologies), 5.5 mM of MgCl_2_, 800 μM of each dNTP, 100 nM of ESFY 16S probe, 900 nM of each ESFY 16S primer, 75 nM of DiSTA 18S probe, 150 nM of each DiSTA18S primer, 10U of Recombinant RNasin(R) RNase Inhibitor (Promega), 1.25 units of AmpliTaq Gold DNA Polymerase, 8 units of M-MLV Reverse Transcriptase and 1 mM DTT.

The triplex reaction was performed adding to the reagents above: 300 nM of each PPV forward primers (P316D and P316M), 400 nM of PPV reverse primer (P241) and 100 nM of probe (PPV-DM). Amplification and detection were performed using an automated ABI PRISM 7000 Sequence Detection System (Life Technologies) in MicroAmp optical 96-well plates.

The following conditions were used for all RT-qPCR assays: 48°C for 30 min, 95°C for 10 min, followed by 40 cycles of 95°C for 15 s and 60°C for 1 min. In all the qPCR assays, nuclease-free Milli-Q water was added in place of reverse transcriptase, using the same amplification conditions.

Relative standard curve method was used to perform relative quantification following Applied Biosystems guidelines. Serial dilutions of crude sap were assessed by RT-qPCR along with the tested samples. The slope (k) of the linear regression curve between logarithmic values of RT-qPCR serial dilutions (y-axis) and Ct values (x-axis) were used to calculate the amplification efficiency (E). The squared regression coefficient after linear regression was also calculated. Samples were normalized to the sample with the highest relative value and expressed as percentages. The standard deviation of the ΔCt is calculated from the standard deviations of the target (s1) and reference (s2) values using the formula: s = (s1^2^ + s2^2^) ^½^.

### DNase treatment

Eight samples positive for ‘*Ca*. P. prunorum’ were prepared with either CTAB or spot method. Samples were divided in two aliquots: one aliquot was treated with 2U of DNase I (New England BioLab) for 2 hours at 37°C; the other one was managed at the same conditions without DNase treatment. Each aliquot was analysed in duplicate in the same plate, with or without reverse transcriptase. DNase treated samples amplified without reverse transcriptase produced no signal proving that DNA was completely digested. Statistical significance was determined by Student’s t-test.

## Results

### Duplex RT-qPCR assay

The designed probe for ESFY detection differs by two mismatches from ‘*Ca*. P. mali’ sequence and three mismatches from ‘*Ca*. P. pyri’ sequence within group 16SrX and shows even more point mutations in comparison with phytoplasma sequences belonging to other groups. The specificity of the selected primers and probes was verified by RT-qPCR using different phytoplasma strains as template. As a result, a fluorescent signal was detected only from ESFY infected samples and no cross-reaction occurred (data not shown).

The endogenous control, named DiSTA 18S, was successfully tested on RNA extracted from several plant species including prunus, pear, apple, strawberry, grape and wheat (data not shown).

All the experiments below for ESFY detection were carried out with the optimized duplex RT-qPCR described in Materials and methods.

### Rapid sample preparation method adjustments

The method optimization was performed on the same apricot phloem-rich samples and empirically selected for the lowest Ct values (specific Ct values are not shown). Among all the tested conditions, 1:5 (w/v) final dilution of the sample in grinding buffer produced the best result. No improvement of Ct values was observed when the additives were tested with the exception of 1mM DTT, which improved the Ct values of 0.5 cycles. Spot method and dilution method were subsequently tested in parallel with the four resuspension solutions. Spot and dilution methods produced the same results with different resuspension solutions: 0,5% Triton X-100 produced no signal; GES-PVPP showed best Ct value followed by GES-PVPP-0,5% Triton X-100 and GES alone with average difference of Ct values of about 1,5 cycles. Finally, 100 μl of GES-PVPP gave slightly better results than 50 μl (average Ct values difference: 0.3 cycles). Therefore, 100 μl of GES-PVPP buffer have been used in the optimized protocol.

Since dilution and spot methods showed similar results, the latter has been chosen for further validation experiments in order to assess storage properties of nylon membrane.

### Validation of the detection method

To evaluate efficiency and reliability of the spot method applied to RT-qPCR we compared it to a well-established method used for the detection of ‘*Ca*. P. prunorum’, such as CTAB extraction coupled with qPCR that therefore use as template only DNA of the phytoplasma.

The two methods were initially tested on 40 plum samples presenting severe ESFY symptoms, collected from the province of Modena (Italy). Ct values were compared and only a slight difference between two methods was observed (S1 Table). The average of the difference between CTAB and spot method Ct values is 0.46 showing that results of spot method coupled with RT-qPCR are slightly better than those of CTAB extraction coupled with qPCR.

These results obtained proved that the sensitivity of the spot method coupled with RT-qPCR is comparable to previous well-established method.

**Fig 1:**
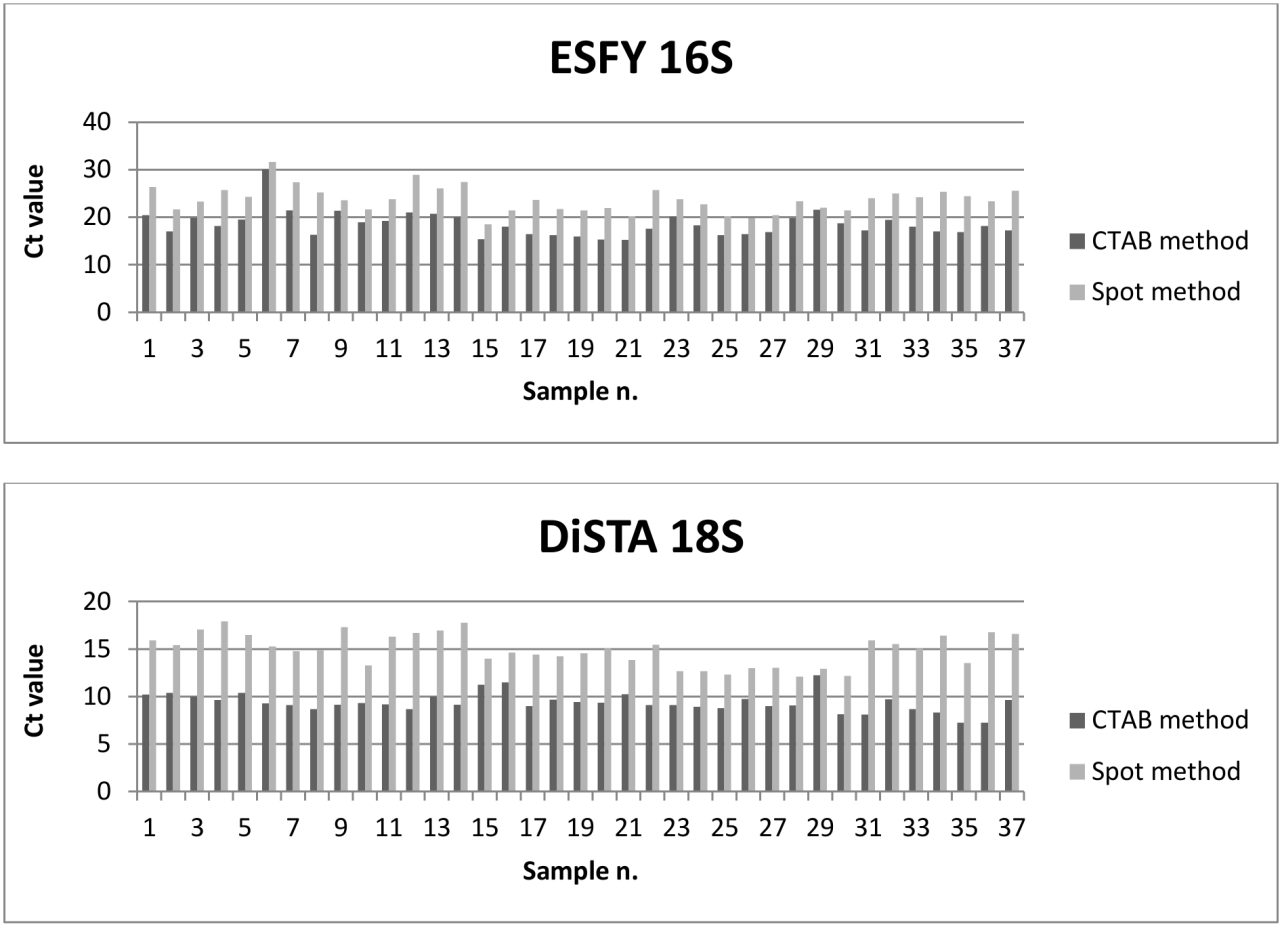
Ct value comparison of CTAB and spot extraction of 37 positive samples after RT-qPCR. The first chart represent ESFY 16S Ct values while the second chart shows DiSTA 18S (plant control) Ct values. On average, the spot method showed a 5.16 (*p* value = 2.8E-12) and 5.58 (*p* value = 1.3E-27) higher Ct values for ESFY 16S and DiSTA 18S assay, respectively.

As a further validation, spot method and CTAB extraction have also been compared using RT-qPCR as detection methods. The two assays were used in parallel to monitor infections in field from spring to autumn 2013. In total, 62 samples of apricot, plum and peach have been tested and both methods produced the same results in terms of detection: 25 healthy and 37 positive samples were found. Both CTAB and spot extraction methods showed no difference in efficacy among the different host plants. A comparison of positive sample Ct values between CTAB and spot methods is reported in Fig 1.

As expected, samples treated with spot method were detected at a higher Ct value than samples extracted with CTAB. On average, the spot method showed Ct values higher of 5.16 (*p* value = 2.8E-12) and 5.58 (*p* value = 1.3E-27) units for ESFY 16S and DiSTA 18S assay, respectively.

Among the 62 samples, 9 positive samples and 1 negative sample extracted with the spot method have been selected to perform relative quantification by RT-qPCR.

**Fig 2:**
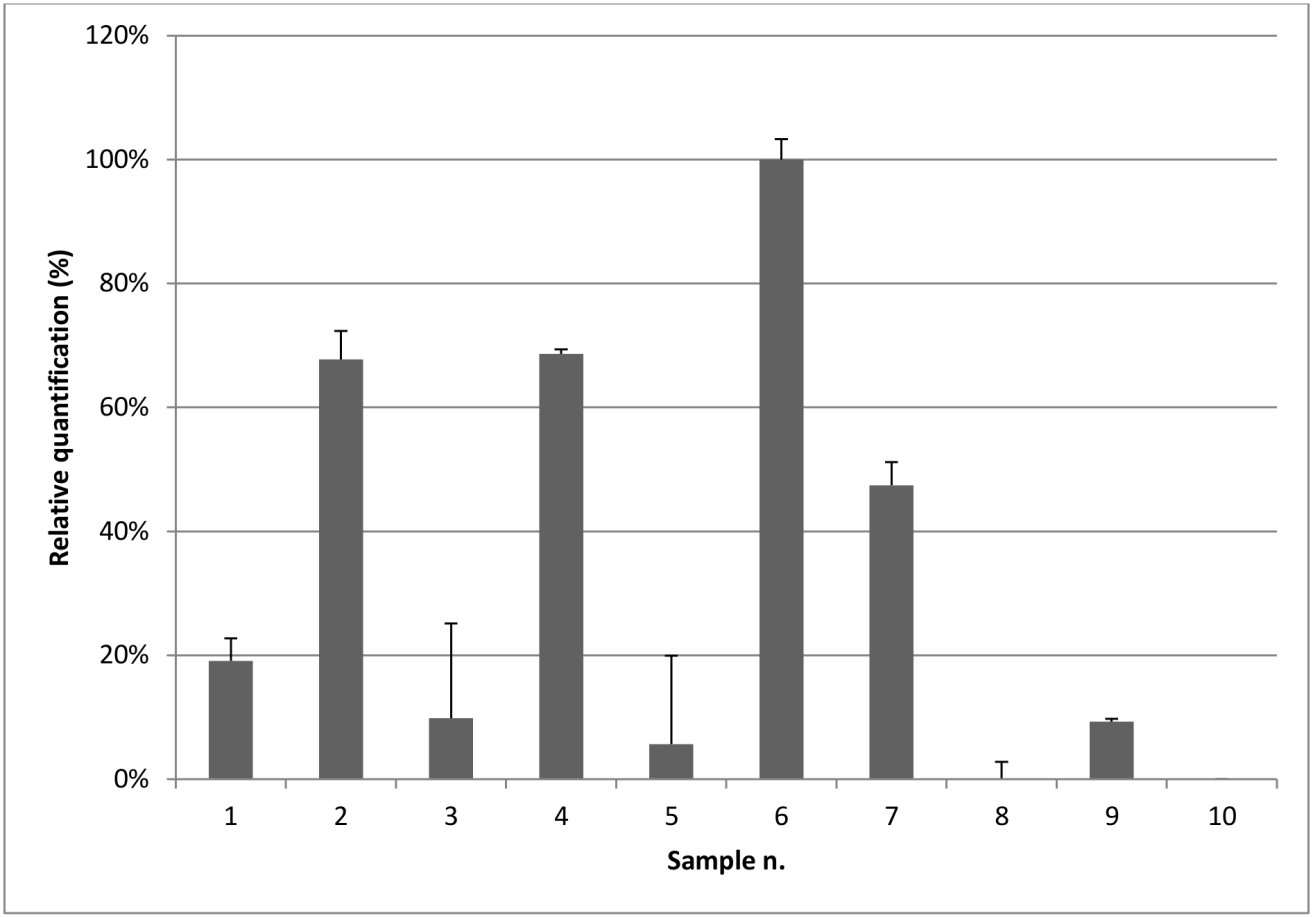
RT-qPCR relative quantification of ESFY on 9 infected samples (1 to 9) and 1 healthy sample (10) extracted with the spot method. Serial dilutions of crude sap were used to calculate the relative quantification using the relative standard curve method. Results from each sample were normalized to the highest value (from sample no. 6) and expressed as percentages.

Fold variation, which is based on the comparison of gene target expression (normalized to the endogenous control) between experimental and control samples, was quantified using the Relative Standard Curve Method. Results are shown in Fig 2.

Serial dilutions were used to calculate k value, the squared regression coefficient (R^2^) and amplification efficiency (E) of ESFY 16S and DiSTA 18S assays. ESFY 16S calculated values are k = -3.5205; R^2^ = 0.9882; E = 0.923316, while DiSTA 18S values are k = -3.6245; R^2^ = 0.9983; E = 0.887557 (S1 Fig).

### DNase Treatment Experiment

**Fig 3:**
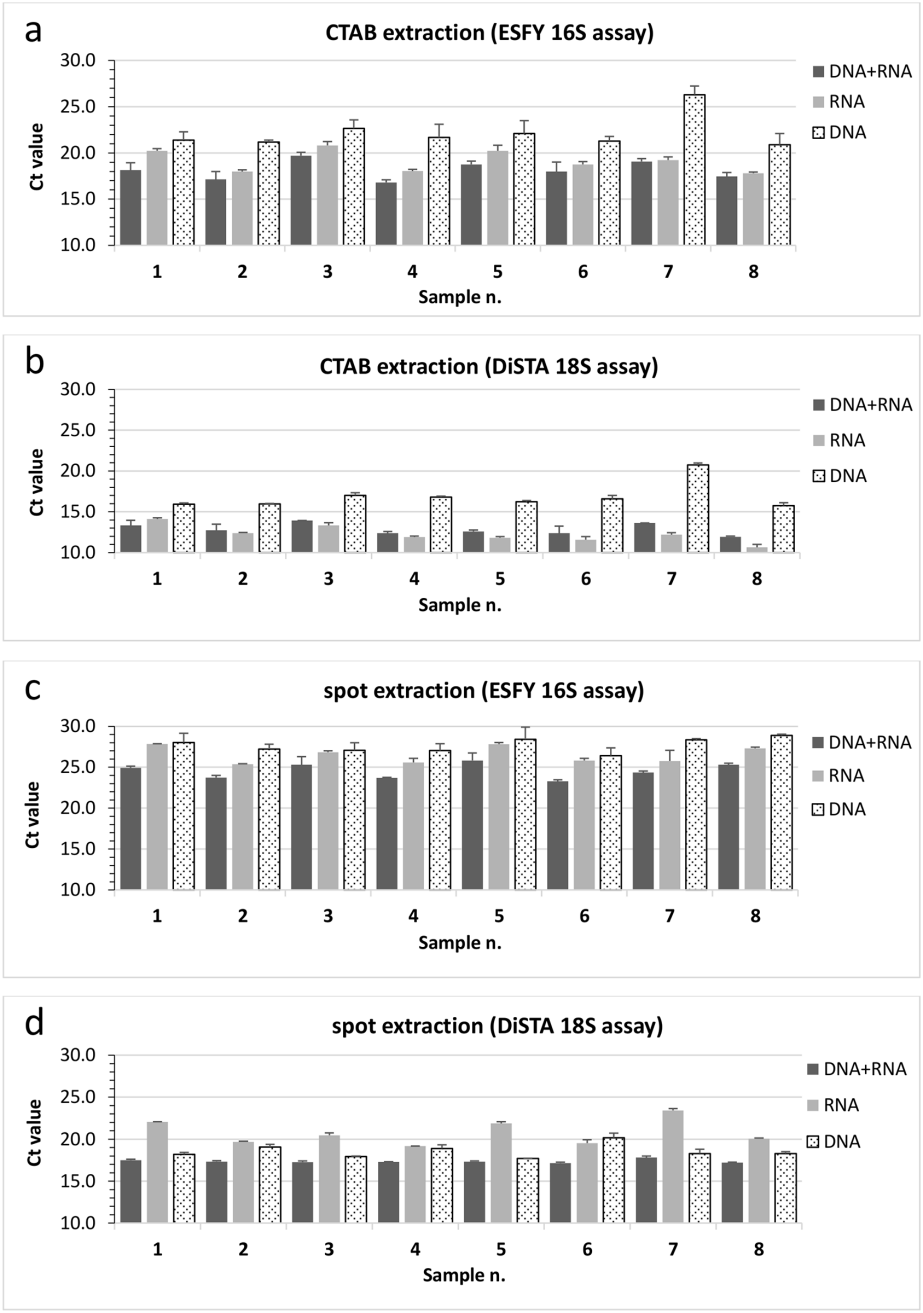
DNA and RNA contribution to detection. Ct value comparison of DNA+RNA (RT-qPCR), DNA alone (qPCR) and RNA alone (RT-qPCR after DNase treatment) on 8 ESFY-positive samples extracted by either CTAB (**a, b**) or the spot method (**c, d**). ESFY 16S assay (**a, c**) detects ‘*Ca*. P. prunorum’ while DiSTA 18S detects the host plant. Samples were: 1 (plum), 2 (apricot), 3 (apricot), 4 (apricot), 5 (plum), 6 (apricot), 7 (plum) and 8 (peach).

In order to investigate the contribution of DNA and/or RNA to qPCR detection, DNase treatment experiments were carried out on eight ESFY-positive samples extracted with both methods. Selected samples were: 1 (plum), 2 (apricot), 3 (apricot), 4 (apricot), 5 (plum), 6 (apricot), 7 (plum) and 8 (peach). Each sample, after CTAB or spot extraction, was divided in two aliquots and only one was treated with DNase. All aliquots were then amplified in duplicate through qPCR with or without a reverse transcriptase step in order to obtain a combined signal from DNA and RNA or a single signal from DNA or RNA, respectively. The resulting Ct values are shown in Fig 3 while Ct value differences are summarized in Table 2. As expected, DNA combined with RNA produced the lowest Ct values in almost all the conditions. CTAB/DiSTA 18S was the only case where RNA outperformed DNA+RNA (+0.6 Ct on average) but the difference was minimal (*p* value = 0.035). Comparing DNA and RNA individual contribution to detection, the latter has always produced lower Ct values except for samples extracted with the spot method and tested for DiSTA 18S assay (+2.2 Ct on average, *p* value = 0.019).

**Table 2:** Averages of the ΔCt values calculated from the DNase treatment experiment (Fig 3). ΔCt values represent the differences between average Ct values of each group, as indicated in the table header: DNA+RNA (RT-qPCR), DNA alone (qPCR) or RNA alone (RT-qPCR after DNase treatment). Analyses have been performed on 8 ESFY-positive samples extracted by either CTAB or the spot method. ESFY 16S assay detects ‘*Ca*. P. prunorum’ while DiSTA 18S detects the host plant. Negative values indicate a gain of Ct value while positive values indicate a delay on Ct values. Student’s t-test was used to determine the statistical significance among the three groups.

extraction method	assay	(DNA+RNA)—DNA		(DNA+RNA)—RNA		RNA—DNA	
		ΔCt value	*p* value	ΔCt value	*p* value	ΔCt value	*p* value
CTAB	ESFY 16S	-4.1	8.22E-05	-1.0	0.003	-3.0	0.002
	DiSTA 18S	-4.0	7.90E-05	+0.6	0.035	-4.6	2.44E-04
spot	ESFY 16S	-3.1	4.12E-06	-2.0	1.32E-05	-1.1	0.008
	DiSTA 18S	-1.2	0.006	-3.4	1.67E-04	+2.2	0.019

### Time course experiment and triplex optimization

Storage property of nylon membrane discs has been evaluated over a period of 4 months on 2 samples (Table 3). After 2 and 4 months, stored spotted discs and fresh spotted discs from stored crude sap produced Ct values similar to Time 0 (*p* values > 0.05). Comparison of spotted membranes stored at room temperature and 4°C showed that both storage conditions gave the same results (*p* values > 0.05).

**Table 3:** Ct values of two samples at time 0 or after 2 months and 4 months of storage at RT or 4°C.

		Time 0	2 months			4 months		
			Frozen Sap	spot RT	spot 4°C	Frozen Sap	spot RT	spot 4°C
**ESFY-16S**	**Sample 1**	26.3±0.6	26.3±0.3	26.2±0.4	26.0±0.4	26.4±0.9	25.2±0.5	25.2±0.1
	**Sample 2**	21.7±0.4	21.8±0.4	22.2±0.8	21.3±0.4	23.5±0.7	21.0±0.7	20.4±0.4
**DiSTA 18S**	**Sample 1**	15.9±1.1	15.8±1.2	18.1±1.1	17.9±09	18.2±1.2	17.3±0.3	17.0±0.6
	**Sample 2**	15.4±0.2	15.7±0.8	16.6±0.4	16.3±0.8	18.1±0.3	16.0±0.7	15.9±0.2

PPV is another severe disease that affects stone fruit plants. Since ‘*Ca*. P. prunorum’ and PPV can affect the same plant species, a triplex RT-qPCR has been set up to simultaneously detect both pathogens and the host plant. Primers and probe for PPV detection designed by Olmos and co-workers [27] were integrated in the duplex RT-qPCR reaction. PPV probe was labelled with FAM in the original paper while here the probe was labelled with VIC fluorophore that emits between FAM and NED spectra (S2a Fig). The optimized reaction produced distinguishable signals from the host plant and from both pathogens (S2b Fig). Repeated assays including negative controls have been performed and no crosstalk between fluorescent probes was detected (data not shown).

## Discussion

The first aim of this paper was to provide an efficient and rapid method for detecting ‘*Ca*. P. prunorum’ in different host plants. Costs and time of the assay are reduced by avoiding nucleic acid extraction, while a good specificity and efficiency are preserved by using TaqMan^®^ probes and adding a reverse transcriptase step to the reaction. A rapid extraction protocol, suitable for large-scale analysis, has been optimised by testing different rapid extraction methods reported prior to this paper. The reliability of the extraction has been confirmed by comparing the optimized spot method to an established method used for ESFY detection, such as CTAB nucleic acid extraction. More than 100 samples were tested during 2013, reporting a similar sensitivity between the two methods of detection. Our validation experiments showed that the lower sensitivity of the spot method which is due to the use of crude sap can be compensated by the introduction of a reverse transcriptase step before the qPCR. To our knowledge this is the first RT-qPCR protocol reported for the detection of ‘*Ca*. P. prunorum’.

This detection protocol is included in the “Diagnostic protocol for ‘*Ca*. P. prunorum’” developed and validated within the Italian project ARNADIA-ARON in order to implement the Council Directive 2002/89/EC on protective measures against the introduction into the Community of organisms harmful to plants or plant products and against their spread within the Community.

RT-qPCR takes advantage of the higher number of copies of 16S rRNA present in active cells compared to the two copies of the 16S rRNA gene. Furthermore, RNA detection provides an evidence of viability and metabolic activity of phytoplasma which is not provided by DNA detection.

In this study, the role of RNA and DNA as template for the reaction has been investigated. Our data showed that combining RNA and DNA detection provides the best sensitivity. Our findings are consistent with those of Margaria and co-workers [15] who demonstrated the advantages of a RT step on Flavescence dorée phytoplasma detection by PCR and qPCR. As expected, a major role seems to be played by RNA. Nevertheless, the nucleic acid contribution to the detection seems to be dependent on specific assay and extraction condition. Indeed, factors like PCR inhibitors, presence of RNase and RNA copy number variation among different organisms must be taken into account. These data suggested that PCR detection methods that are able to amplify both RNA and DNA are likely to provide the best sensitivity among different organisms and extraction conditions.

The assay specificity, improved by the use of MGB conjugated probes [28], was rigorously tested and no cross-reaction with other phytoplasma strains was detected. Relative quantification of phytoplasma is facilitated by an endogenous control specific for plant 18S rRNA and an example of its feasibility was provided. Efficiency of both ESFY 16S and DiSTA 18S assays and guidelines for a correct relative quantification were also presented.

Concerning the optimization of the rapid sample preparation method we observed no significant difference between the use of nylon membrane as support and simple dilution of sap in tubes. This suggests that the buffer and the dilution factor are the main elements that contribute to the RT-qPCR feasibility. Nevertheless, we believe that the introduction of a nylon support may help with the preservation of samples over long time and reduce the risk of PCR contaminations. Indeed, nylon membranes proved to be an efficient way to store, transport or ship samples without the need of refrigeration.

Finally, a triplex RT-qPCR assay has been developed to simultaneously detect ‘*Ca*. P. prunorum’ and PPV in *Prunus*, as these diseases may occur in the same plant species. Along with ESFY, PPV is one of the most detrimental and important pathology of stone fruit trees [27]. PPV is a virus with a linear single stranded RNA genome and is localized within the phloem, like phytoplasmas. Therefore, sampling can be done from the same tissue and RT-qPCR can be performed on the same crude sap, without the need to extract nucleic acids. The described triplex assay could decrease costs, time and labour of stone fruit tree screening in areas susceptible to both pathologies. Nevertheless, further experiments are necessary for a proper validation of this assay. Indeed, the triplex assay was not tested with naturally infected samples. Similar to other tree fruit viruses, PPV concentration can be low at certain times of the year and unevenly distributed in trees that are newly infected [29]. It also remains to be determined how the concentration of one target influences the qPCR efficiency for the other. Thus, a validation with field samples would be necessary to prove that a parallel screening is feasible.

## Supporting Information

S1 Fig Visual representation (a) and plotted chart (b) of serial dilutions RT-qPCR assay used for relative quantification. Serial dilutions were used to calculate k value, the squared regression coefficient (R^2^) and amplification efficiency (E) of ESFY 16S (in red) and DiSTA 18S (in green) assays. Two technical replicates were performed and resulting Ct values were averaged. ESFY 16S calculated values are k = -3.5205; R^2^ = 0.9882; E = 0.923316, while DiSTA 18S values are k = -3.6245; R^2^ = 0.9983; E = 0.887557. ΔCt values represent the difference between target Ct values and reference Ct values are in yellow in S1B Fig. The resulting equation of the regression line for ΔCt values is y = 0.235x + 6.9687. As the slope value (0.235) is higher than 0.1, ΔΔCt method cannot be applied and serial dilutions must be included every time that relative quantification is required.
(PDF)

S2 Fig Design of the triplex RT-qPCR to simultaneously detect ‘*Ca*. P. prunorum’ (FAM, in red) PPV (VIC, in blue) and the host plant (NED, in green). Emission spectrum of different fluorogenic probes (a) and an example of the resulting triplex RT-qPCR (b) are shown.
(PDF)

S1 Table Comparison of the Ct values obtained with CTAB extraction method coupled with qPCR and spot extraction method coupled with RT-qPCR on 40 plum samples affected by ESFY. The difference between Ct values of CTAB method with qPCR and Ct values of spot method with RT-qPCR was calculated for each sample.
(DOCX)
