# Supplementary figures and images for: A Rapid Protocol of Crude RNA/DNA Extraction for RT-qPCR Detection and Quantification of 'Candidatus Phytoplasma prunorum'
Source: PLoS One. 2016 Jan 7;11(1):e0146515. doi: 10.1371/journal.pone.0146515 (PMC4704776; doi:10.1371/journal.pone.0146515)

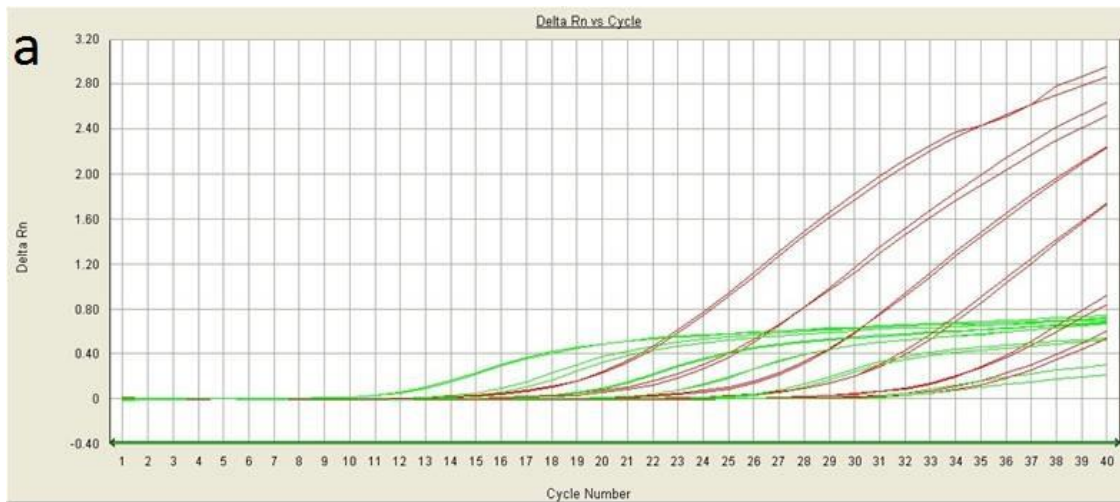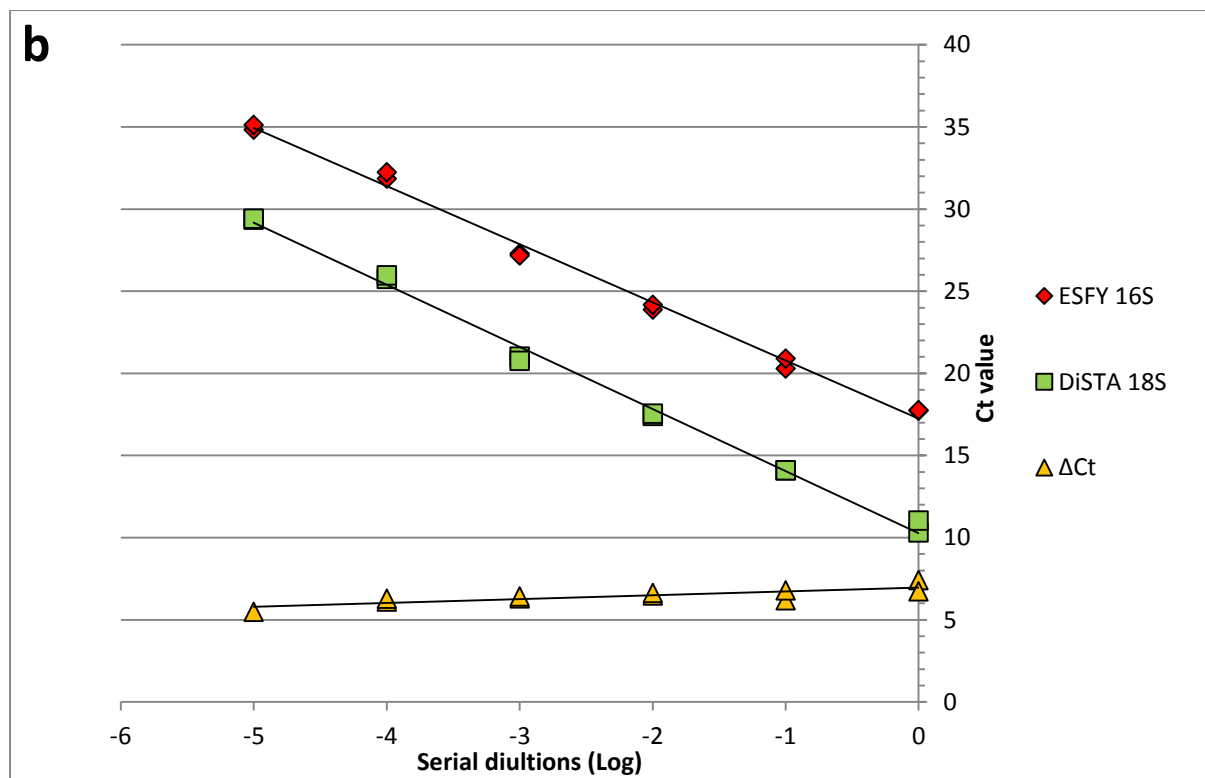

Supplement: S1 Fig — Serial dilutions were used to calculate k value, the squared regression coefficient (R2) and amplification efficiency (E) of ESFY 16S (in red) and DiSTA 18S (in green) assays. Two technical replicates were performed and resulting Ct values were averaged. ESFY 16S calculated values are k = -3.5205; R2 = 0.9882; E = 0.923316, while DiSTA 18S values are k = -3.6245; R2 = 0.9983; E = 0.887557. ΔCt values represent the difference between target Ct values and reference Ct values are in yellow in S1B Fig. The resulting equation of the regression line for ΔCt values is y = 0.235x + 6.9687. As the slope value (0.235) is higher than 0.1, ΔΔCt method cannot be applied and serial dilutions must be included every time that relative quantification is required. (PDF) [file pone.0146515.s001.pdf]

**a**

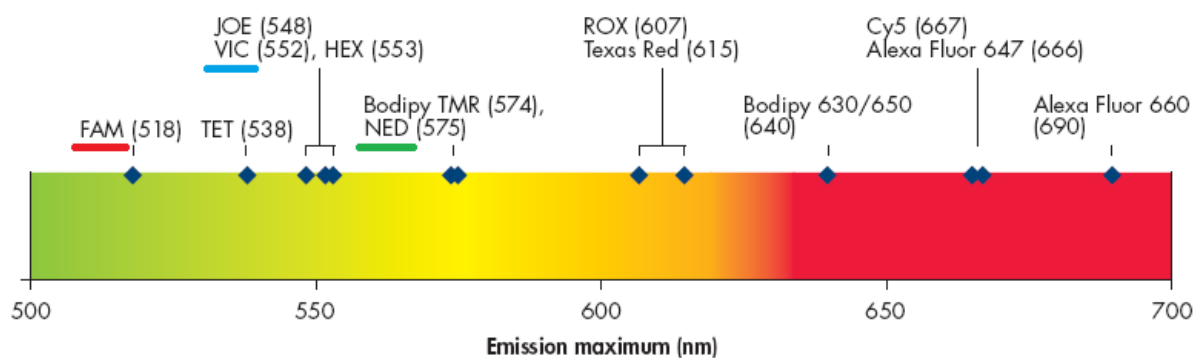

**b**

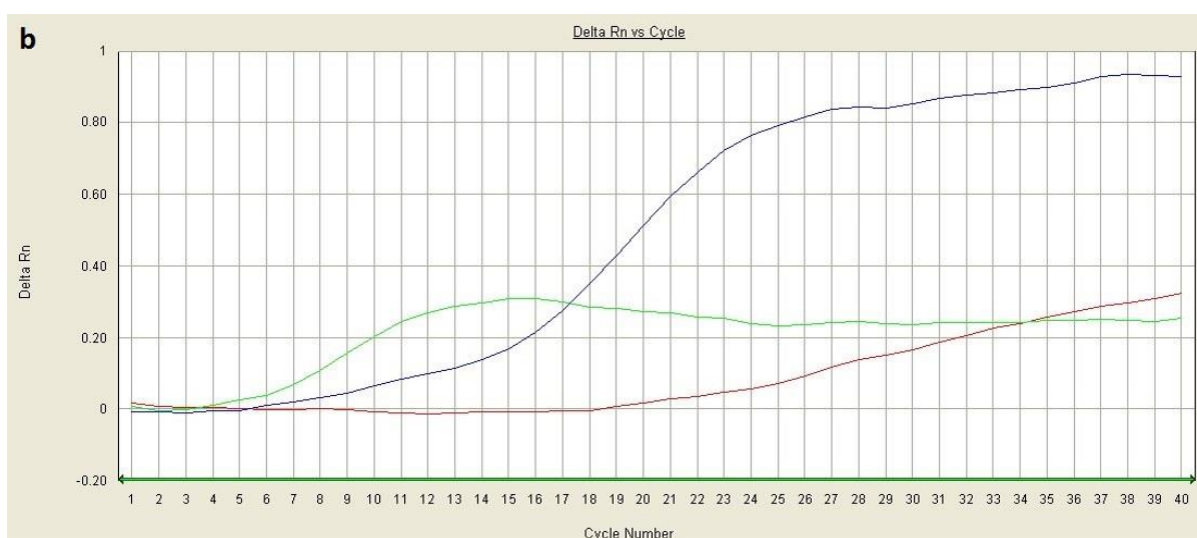

Supplement: S2 Fig — Emission spectrum of different fluorogenic probes (a) and an example of the resulting triplex RT-qPCR (b) are shown. (PDF) [file pone.0146515.s002.pdf]
