# Supplementary material for: A Rapid Protocol of Crude RNA/DNA Extraction for RT-qPCR Detection and Quantification of 'Candidatus Phytoplasma prunorum'
Source: PLoS One. 2016 Jan 7;11(1):e0146515. doi: 10.1371/journal.pone.0146515 (PMC4704776; doi:10.1371/journal.pone.0146515)
Supplement: S1 Table — The difference between Ct values of CTAB method with qPCR and Ct values of spot method with RT-qPCR was calculated for each sample. (DOCX) [file pone.0146515.s003.docx]

| **Sample** | **CTAB method and qPCR (Ct value)** | **Spot method and RT-qPCR (Ct value)** | **CTAB - SPOT (Ct value)** |
| --- | --- | --- | --- |
| A1 | 23.45 | 22.10 | 1.35 |
| A2 | 25.09 | 22.48 | 2.61 |
| A3 | 21.94 | 19.64 | 2.30 |
| A4 | 25.00 | 24.20 | 0.80 |
| A5 | 26.88 | 23.16 | 3.72 |
| A6 | 24.67 | 22.29 | 2.38 |
| A7 | 24.60 | 22.18 | 2.42 |
| A8 | 23.09 | 22.44 | 0.65 |
| A9 | 23.00 | 21.43 | 1.58 |
| A10 | 21.01 | 20.44 | 0.58 |
| A11 | 22.59 | 24.31 | -1.72 |
| A12 | 21.82 | 26.81 | -4.99 |
| A13 | 23.48 | 23.40 | 0.08 |
| A14 | 22.04 | 24.60 | -2.56 |
| A15 | 23.65 | 23.58 | 0.07 |
| A16 | 21.84 | 22.76 | -0.92 |
| A17 | 24.48 | 23.24 | 1.24 |
| A18 | 21.49 | 20.05 | 1.44 |
| A19 | 29.72 | 21.24 | 8.48 |
| A20 | 22.80 | 23.31 | -0.51 |
| A21 | 31.00 | 29.40 | 1.60 |
| A22 | 24.41 | 27.03 | -2.62 |
| A23 | 26.31 | 26.74 | -0.43 |
| A24 | 25.08 | 28.38 | -3.30 |
| A25 | 25.72 | 27.23 | -1.51 |
| A26 | 25.88 | 26.53 | -0.65 |
| A27 | 27.17 | 25.95 | 1.22 |
| A28 | 30.00 | 29.55 | 0.45 |
| A29 | 29.67 | 29.47 | 0.21 |
| A30 | 32.85 | 26.25 | 6.59 |
| A31 | 26.18 | 29.17 | -2.98 |
| A32 | 25.86 | 30.93 | -5.07 |
| A33 | 26.85 | 26.15 | 0.70 |
| A34 | 26.78 | 28.91 | -2.13 |
| A35 | 27.28 | 27.47 | -0.19 |
| A36 | 26.61 | 23.94 | 2.68 |
| A37 | 32.36 | 27.97 | 4.39 |
| A38 | 26.47 | 24.81 | 1.66 |
| A39 | 24.05 | 26.29 | -2.24 |
| A40 | 26.93 | 26.08 | 0.85 |
| Average | 25.50 | 25.05 | 0.46 |
